# Hyperbranched Thermosensitive Polymer-AuNP Composite Probe for Temperature Colorimetric Detection

**DOI:** 10.3390/s24227124

**Published:** 2024-11-06

**Authors:** Huidong Li, Yao Zhou, Junqi Gu, Wenjie Zhong, Xinlong Li, Xunyong Liu, Zhuhui Qiao, Yi Liu

**Affiliations:** 1 Shandong Laboratory of Yantai Advanced Materials and Green Manufacturing, Yantai 264006, China; lhd6661998@163.com (H.L.); zwj15684139786@126.com (W.Z.); jingxin1028@163.com (X.L.); 2 School of Chemistry and Materials Science, Ludong University, Yantai 264025, China; lud_zhouyao1999@163.com (Y.Z.); gjqxy999@163.com (J.G.)

**Keywords:** temperature-sensitive polymers, gold nanoparticles, colorimetric probes, visual temperature detection, flexible sensors

## Abstract

Temperature detection is particularly important in the medical and scientific fields. Although there are various temperature detection methods, most of them focus on broad temperature detection, and basic research in specific fields, especially the detection of subtle temperature changes (32–34 °C) during wound infection, is still insufficient. For this purpose, a novel colorimetric temperature sensing probe is designed in this paper, which can quickly and intuitively respond to small temperature changes within a specific range through color changes. In this paper, hyperbranched polyethyleneimine (HPEI) was modified by isobutyrylation to prepare hyperbranched temperature-sensitive polymer (HPEI-IBAm). And it was combined with gold nanoparticles (AuNPs) prepared by a sodium citrate reduction method to construct an HPEI-IBAm-AuNP colorimetric probe. The probe exhibits excellent stability, even at salt concentrations of up to 12 g/L, thanks to the abundant amino functional groups and the large steric hindrance effect unique to HPEI-IBAm. In particular, the temperature detection range of the probe is precisely locked within 32–34 °C, enabling it to respond quickly and accurately to small temperature changes of only 2 °C. This feature is perfectly suited to the practical needs of temperature detection in infected wounds. The linear fitting coefficient of the temperature response is as high as 0.9929, ensuring the accuracy of the test results. The detection performance of the probe remained highly consistent over 10 cycles, fully proving its excellent reusability and durability. In addition, a flexible colorimetric sensor was prepared by combining the probe with polydimethylsiloxane (PDMS) film. This sensor is capable of rapidly detecting human skin temperature in real time, achieving an accuracy of 99.07% to 100.61%. It can provide a possible solution to the challenges of delayed and difficult temperature detection caused by different body parts and uneven surfaces, among others. This demonstrates its extensive practical value and potential, and it is expected to be further applied in the monitoring of wound infections.

## 1. Introduction

Temperature, as a macroscopic manifestation of the thermal properties of matter, plays an indispensable and important role in a wide array of fields such as industrial production, the application of medical devices, and scientific research [1,2]. To date, a variety of sensor technologies have emerged for temperature detection, such as thermocouples [3,4], liquid-filled glass thermometers [5], and optical sensors [6], which are widely used for their high sensitivity and accuracy. However, they mostly focus on broad temperature-range monitoring, and their relevance and accuracy are still insufficient for some specific areas, especially for application scenarios that require the precise capture of small temperature changes (e.g., infected wound temperature monitoring, typically in the range of 32–34 °C). In view of this, the development of a simple, rapid, highly sensitive, highly stable, and accurate temperature detection method tailored for specific fields and temperature ranges is particularly urgent and important.

Wound healing is a complex process [7,8]. One of the most serious problems that often occurs during wound healing is wound infection, which can cause redness, swelling, heat, pain, sepsis, and even the amputation of the affected limb [9,10]. The temperature in the wound environment serves as an important indicator of wound infection [11,12]. The main sensors reported as used for wound temperature monitoring are electrochemical devices such as thermistors or thermoelectric-type sensors [13], micromechanical sensors, [14] or voltametric sensors [15]. Although these electrochemical sensors exhibit high accuracy and sensitivity [16], they are primarily used by professionals and are inconvenient to operate. In addition, electrochemical sensors have poor mechanical properties [17], lacking the ability to bend. Due to the differences in parts of the human body and uneven surfaces, they do not adhere closely to the skin, which can easily result in detection hysteresis, difficulties in detection, and other phenomena. Colorimetric detection offers a potential solution to these limitations due to its rapid feedback and easy quantification of the signal. However, its current applications are mainly focused on easily regulatable ion detection, and its applications in temperature detection are still limited. Moreover, the temperature detection range is relatively broad and has not been effectively combined with advanced materials such as polymer film, which limits its practical application in the field of human skin temperature detection. Therefore, it is particularly important to develop a flexible colorimetric probe that matches the temperature range of wound infection (32–34 °C) and can effectively fit all parts of the human body.

Colorimetric temperature sensors are mainly constructed by combining dyes or noble metal nanoparticles (NMNPs) with temperature-responsive materials [18]. These materials show visible color due to strong absorption in the visible light region based on localized surface plasmon resonance (LSPR). The principle is based on the shift in the absorption spectrum, which is caused by changes in the energy level difference of the system after recognition, or a color change resulting from a large change in the absorption coefficient [19,20]. The most commonly used temperature-responsive material is linear temperature-sensitive poly(N-isopropylacrylamide) (PNIPAM) [21,22]. For example, Zhu et al. [23] prepared temperature-sensitive AuNPs using sulfhydryl-modified PNIPAM, with a low critical solution temperature (LCST) of 28.4 °C, and the phase transition process occurred between 25 and 30 °C, accompanied by a color change from red to purple. Cha et al. [24] conducted a detailed review of hybrid microgels loaded with AuNPs or silver nanoparticles (AgNPs) based on PNIPAM or its copolymers, all of which exhibited temperature-responsive properties along with the loaded NMNPs. Lin et al. [25] used star-shaped thermosensitive poly(acrylic acid-b-N-isopropylacrylamide) (PAA-b-PNIPAM) as a single-molecule nanoreactor and prepared PAA-b-PNIPAM-encapsulated AuNPs through in situ reduction. The experiment showed that as the temperature increased from 20 to 50 °C, the LSPR of the AuNPs shifted by only about 10 nm in the absence of excess free PNIPAM, and the solution color did not change significantly. This was attributed to the lack of free PNIPAM that could act as a physical crosslinking agent and could not cause aggregation between neighboring AuNPs. When additional excess PNIPAM was added, the system showed a noticeable red shift and color change. Differently, AuNPs stabilized by thermosensitive dendritic polymers could show a significant red shift and color change during a temperature rise, mainly because the multi-functional groups of dendritic polymers could play a good physical crosslinking role. Unfortunately, current temperature-sensitive AuNPs usually exhibit a wide temperature response range (greater than 5 °C) and are not sensitive enough for the accurate detection of infected-wound temperature, which may vary by only 2 °C, as shown in Table S1. Moreover, not all substrates combined with probes can effectively ensure the appearance of color change for the detection object.

Therefore, in view of the above problems, this study takes advantage of the special topological structure of HPEI-IBAm, the sensitive temperature-response properties of the material, and the advantages of AuNPs in colorimetric detection applications, along with the synergistic effect between them, to construct a visual sensing system. The system, which uses polydimethylsiloxane film as the support, has strong affinity to the skin and is capable of responding to changes in the temperature of infected wounds, enabling real-time temperature monitoring. Consequently, the problems such as the wide response range of current temperature detection probes, their weak adhesion to the skin, and their inability to be applied to different parts of the human body can be addressed. The probe can quickly and intuitively respond to subtle temperature changes within a specific range, of 32–34 °C, through color changes, with a linear fitting coefficient of 0.9929, providing a possibility for application in wound infection monitoring. At the same time, the probe has also shown excellent performance in practical applications, working stably in complex environments while maintaining a high degree of detection accuracy and reliability, with an accuracy rate ranging from 99.07% to 100.61%. In summary, the developed flexible visual probe holds great potential in practical applications and is expected to be further applied to the monitoring of wound infections.

## 2. Experimental Section

### 2.1. Materials and Apparatus

Hyperbranched polyethyleneimine with an *M*_n_ = 10^4^ g/mol was purchased from Aldrich (Missouri, USA.). Chloroauric acid (HAuCl_4_) was purchased from Tianjin Jiaye Precious Metals Technology Company (Tianjin, China). Trichloromethane (CHCl_3_), triethylamine (C_6_H_15_N), isobutyric anhydride (C_8_H_14_O_3_), potassium carbonate (K_2_CO_3_, AR), and other reagents were purchased from Sinopharm Chemical Reagent Co., Ltd (Shanghai, China).

Ultraviolet-visible (UV–vis) absorption spectra were recorded using a T700 UV–vis spectrophotometer (Purkinje General, Beijing, China). The morphology and size of AuNPs and HPEI-IBAm-AuNPs were investigated using transmission electron microscopy (TEM, Libra 200, Zeiss, Oberkochen, Germany) and dynamic light scattering (DLS, Malvern, UK). The elemental composition of the materials was analyzed by X-ray photoelectron spectroscopy (XPS, Thermo Scientific, Thermo Scientific, USA). A nuclear magnetic resonance spectrometer (Bruker-II 500 MHz, Brukey, Massachusetts, USA) was used for analysis to confirm if HPEI-IBAm was successfully prepared and to calculate its acylation degree.

### 2.2. Preparation of AuNPs

AuNPs were prepared by reducing chloroauric acid (HAuCl_4_·4H_2_O) solution using sodium citrate as the reducing agent, as shown in Equation (1). The preparation process was as follows: First, 3.9 mL of a 4.86 mM HAuCl_4_·4H_2_O solution was measured and added to 78.3 mL of deionized water, which was then heated to boiling. Subsequently, 6 mL of a 0.035 M sodium citrate solution was added, and the solution was kept boiling while being vigorously stirred. AuNPs were obtained when the solution turned wine red, and were then refrigerated for later use.

(1) $${2AuCl}_{3}+3{{(}^{-}{OCOCH}_{2})}_{2}C\left(OH\right){COO}^{-}\overset{∆}{\to }{2Au}^{0}+3{{(}^{-}{OCOCH}_{2})}_{2}CO+3{CO}_{2}+{3H}^{+}+{6Cl}^{-}$$

### 2.3. Preparation of HPEI-IBAm-AuNPs

**Scheme 1:**
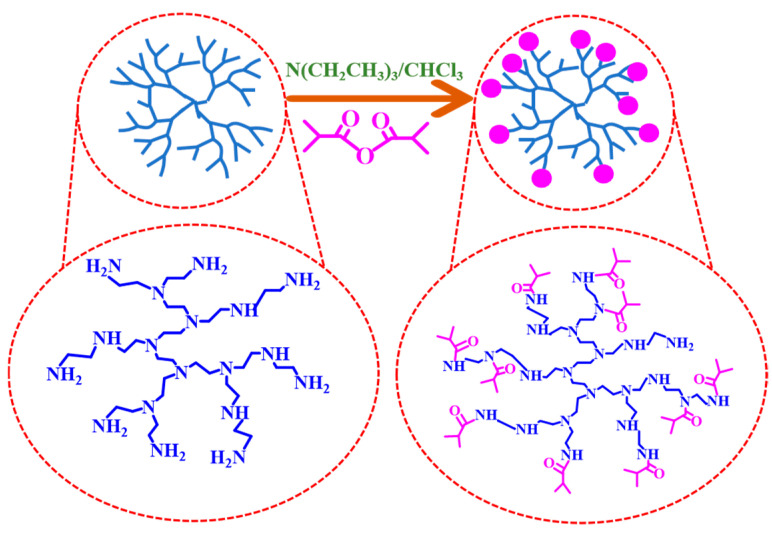
HPEI-IBAm preparation flowchart.

HPEI-IBAm was prepared through the reaction of HPEI and C_8_H_14_O_3_ in the presence of redistilled triethylamine as an acid acceptor, as shown in Scheme 1. Firstly, 2 g of HPEI was weighed and dissolved in 20 mL of chloroform. Then, 3.02 g of redistilled triethylamine and 4.43 g of isobutyric anhydride were added under the condition of an ice-bath. The reaction was carried out under nitrogen protection at room temperature for 24 h. Subsequently, the system was heated to 70 °C and refluxed for 2 h to ensure complete reaction. At the end of the reaction, the solution was cooled to room temperature and filtered under vacuum to remove insoluble salts, followed by distillation to remove volatile components and obtain a viscous crude product. It was dissolved in 40 mL of methanol, and 1 g of potassium carbonate solid was added. The mixture was stirred at room temperature for 5 h to neutralize any possible residual acid. After centrifugation, the solution was concentrated to 15 mL and dialyzed for 72 h using a benzoylated cellulose dialysis membrane with a cut-off molecular weight of 1000 Da. The dialyzed solution was then filtered through a 0.45 μm oil membrane, and the methanol was evaporated. The product was subsequently dried under vacuum for 24 h to obtain HPEI-IBAm with an acylation degree of 80%. A series of HPEI-IBAm samples with different acylation degrees were prepared under the same conditions by varying the amounts of redistilled triethylamine and isobutyric anhydride. Finally, the prepared HPEI-IBAm was complexed with an AuNPs solution to obtain the HPEI-IBAm-AuNPs probe.

### 2.4. Preparation of Polydimethylsiloxane Film-Based Flexible Probes

Dow Corning SYLGARD184 elastomer and crosslinker were mixed at a mass ratio of 10:1. The mixture was then ultrasonically defoamed for 30 s and degassed in a vacuum for 30 min. Using a 400 nm film scraper, the mixture was scraped on a soapy water-treated glass substrate (10.0 cm × 5.0 cm) [26]. The PDMS layer on the glass substrate was cured at 60 °C for 2 h and then cut into 3 cm × 4 cm squares. After overlapping the two films, the surrounding edges were coated with uncured elastomer and crosslinker for curing and sealing, leaving a small hole for injection. The HPEI-IBAm-AuNP probe was injected through this hole, followed by additional curing and sealing to prepare the flexible temperature colorimetric probe.

### 2.5. Optimization of Detection Conditions

The effects of HPEI-IBAm acylation degree, HPEI-IBAm concentration, AuNP concentration, and pH on the detection system were systematically investigated. For the screening of HPEI-IBAm with different acylation degrees, AuNPs and HPEI-IBAm were added to the system, with the volume of the system fixed at 2 mL. The concentrations of AuNPs and HPEI-IBAm in the system were set to 0.225 mM and 2 mg/mL, respectively. By comparing the detection performance of the systems, the optimal acylation degree of HPEI-IBAm for the preparation of HPEI-IBAm-AuNP probes was determined. Similarly, different concentrations of HPEI-IBAm or AuNPs were added to the detection system to investigate their responses to temperature changes, thereby optimizing their respective optimal concentrations. Regarding the impact of pH, it is noteworthy that acidity tends to elevate the cloud point of the system and may even cause it to disappear, whereas alkalinity has the opposite effect, narrowing the interval between clear and turbid states. In a 2 mL detection system where the concentrations of AuNPs and HPEI-IBAm were maintained at 0.45 mM and 2.25 mg/mL, respectively, the pH value was adjusted, and its temperature responsiveness was investigated to determine the optimal pH for the system.

### 2.6. Salt Stability of HPEI-IBAm-AuNP Probes

In seven samples, the concentrations of HPEI-IBAm and AuNPs were fixed at 2.25 mg/mL and 0.45 mM, respectively. To each mixed system, 1 mL of NaCl solution with mass concentrations of 0, 2, 4, 6, 8, 10, and 12 g/L was added, respectively, followed by the addition of deionized water to adjust the volume to 2 mL. By observing and recording the changes in the color and UV–vis spectra of the probes, the salt tolerance stability of the probe system was evaluated.

### 2.7. The Detection Sensitivity and Cycling Stability of the HPEI-IBAm-AuNP Probe

To thoroughly investigate the sensitivity of the HPEI-IBAm-AuNP probe for temperature detection and its cycling performance, we designed the following experimental scheme: Firstly, we prepared a probe system consisting of AuNPs at a concentration of 0.45 mM combined with HPEI-IBAm (65% degree of acylation) at 2.25 mg/mL. Subsequently, the temperature of the probe system was gradually changed, and changes in the color and UV–vis absorption spectrum of the system were meticulously recorded and observed. The data obtained were fitted to establish the relationship between wavelength and temperature. In addition, to evaluate the cycling performance of the probes, 10 ramp-up/down cycling experiments were conducted to comprehensively assess whether the probes could maintain stable and reliable detection performance after multiple uses by comparing and analyzing data before and after the cycling.

### 2.8. Practical Application of HPEI-IBAm-AuNP Probes for Temperature Detection

Under optimal experimental conditions, the concentrations of HPEI-IBAm and AuNPs, as well as the pH of the system, were fixed, and the probe was sealed in a PDMS membrane to prepare a flexible colorimetric sensor. The performance of this sensor was subsequently evaluated for its potential applications in skin temperature detection and environmental temperature detection.

## 3. Results and Discussion

**Scheme 2:**
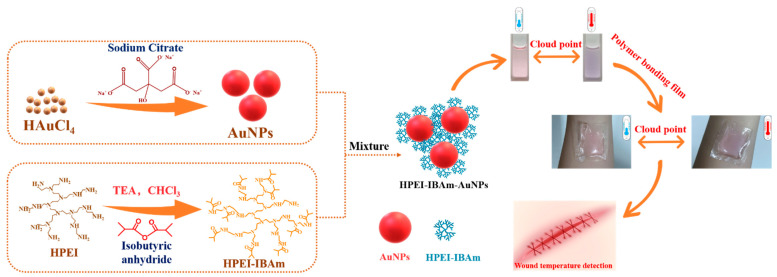
Preparation of HPEI-IBAm-AuNPs and detection mechanism for skin temperature.

As shown in Scheme 2, HPEI was modified by isobutyl acylation to yield the hyperbranched temperature-sensitive polymer, specifically HPEI-IBAm. HPEI-IBAm was combined with AuNPs prepared by a sodium citrate reduction method to construct a HPEI-IBAM-AuNP visual probe. Subsequently, the HPEI-IBAm-AuNPs were infused into PDMS membranes to fabricate flexible colorimetric sensors. Based on the interaction between hyperbranched thermosensitive polymers and AuNPs, the probe undergoes a reversible aggregation and disaggregation of nanoparticles accompanied by a change in color as the temperature varies. Thus, a novel temperature–visual detection method was constructed.

### 3.1. Characterization of HPEI-IBAm-AuNPs

**Figure 1:**
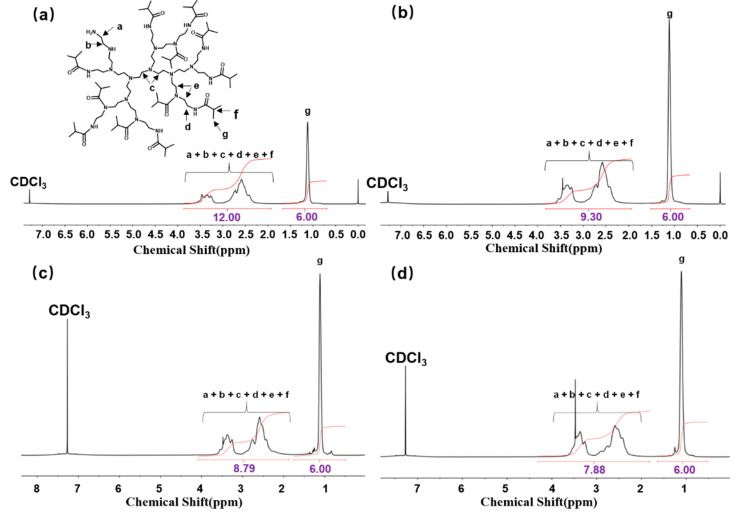
The ^1^H NMR spectra of HPEI-IBAm with the following degrees of acylation: (**a**) 50%, (**b**) 65%, (**c**) 70%, and (**d**) 75% in CDCl_3_.

According to the analysis of the ^1^H NMR spectrum in Figure 1, the characteristic signal of the methyl protons in the isobutyramide group appears at 1.09 ppm (g in Figure 1a), demonstrating the successful isobutyl acylation of the amino groups on the HPEI molecules. Furthermore, new broad peak absorptions appear between 3.0 and 3.7 ppm, which are ascribed to the protons on the methylene group near the N atom in the amide group (d and e in Figure 1a) and the submethyl protons in the isobutyramide group (f in Figure 1a). The integral area I_g_ of g in Figure 1 represents the area of the six hydrogens of the two methyl groups in the isobutyramide group, so that I(g)/6 can be expressed as the number of isobutyramide groups. I(f) represents the area of the submethyl protons in isobutyramide group, which is equal to the number of isobutyramide groups (I(g)/6). I(a + b + c + d + e + f) denotes the sum of the number of protons on the methylene groups and the number of protons on the secondary carbons in the isobutyramide group. This can be interpreted as every two methylene groups are connected to one amino group, so (I(a + b + c + d + e + f) − I(f))/4, i.e., (I(a + b + c + d + e + f) − I(g)/6)/4, can represent the number of all the amino groups in HPEI. The ratio of primary, secondary, and tertiary amines in the HPEI macromolecule can be calculated by ^13^C NMR spectroscopy. For the HPEI10K sample, the ratio of primary, secondary, and tertiary amines is approximately 33:40:27 [27]. Based on this ratio, we utilized Equation (2) to calculate the degree of substitution of the primary and secondary amines (0.73 of all amine groups) by isobutyrylation in HPEI10K. Through computational verification, we successfully prepared HPEI-IBAm with acylation degrees of 50%, 65%, 70%, and 75%.

(2) D(HPEI10K)=(4[I(g)/6]/[I(a+b+c+d+e+f)−I(g)/6])/0.73

**Figure 2:**
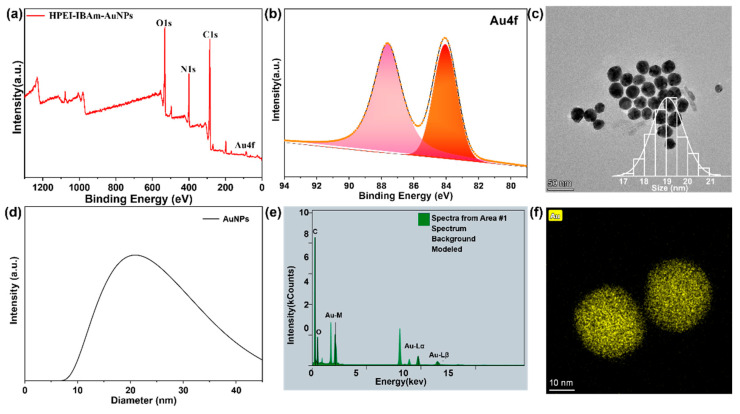
XPS full spectrum of HPEI-IBAm-AuNPs (**a**) and high-resolution XPS image of Au (**b**); transmission electron microscopy (**c**) of HPEI-IBAm-AuNPs and its dynamic light scattering particle size image (**d**); EDS energy spectra (**e**), and EDS photographs (**f**) of AuNPs.

To verify the success of the preparation of HPEI-IBAm-AuNPs, we analyzed their composition using X-ray photoelectron spectroscopy (XPS). From the full XPS spectrum presented in Figure 2a, we can clearly identify the elemental peaks of C, N, and O attributed to HPEI-IBAm and the characteristic elemental peaks of Au attributed to AuNPs. This intuitive evidence strongly indicates that the interaction between the polymer matrix and the AuNPs has occurred to form a stable composite system. As shown in Figure 2b, the XPS high-resolution spectrum of Au can be assigned to two peaks, Au4f_7/2_ and Au4f_5/2_, with binding energies of 84.06 and 87.62 eV, respectively, which is consistent with the electronic energy spectrum of the AuNPs prepared by Li et al. [28], demonstrating that the AuNPs of Au^0^ were successfully prepared from Au^3+^. The resulting complexes were characterized by transmission electron microscopy (TEM) to qualitatively assess the interaction of the polymer with the AuNPs. As shown in Figure 2c, the particle size of AuNPs was determined to be about 19 nm, whereas the average particle size measured by the dynamic light scattering (DLS) technique in Figure 2d was 21 nm, which is larger than the TEM results. This is because DLS measures the hydrodynamic diameter of nanoparticles dispersed in water with a loose nanostructure [27,29]. In addition, the composition of the nanoparticles was further confirmed by energy-dispersive X-ray spectroscopy (EDS) analysis of the probe AuNPs (Figure 2e,f), which clearly demonstrated the uniform distribution of Au elements. In summary, not only were AuNPs successfully prepared, but the effective combination with the temperature-sensitive polymer was also achieved.

### 3.2. Optimization of Experimental Conditions for Detection Temperature of HPEI-IBAm-AuNPs

**Figure 3:**
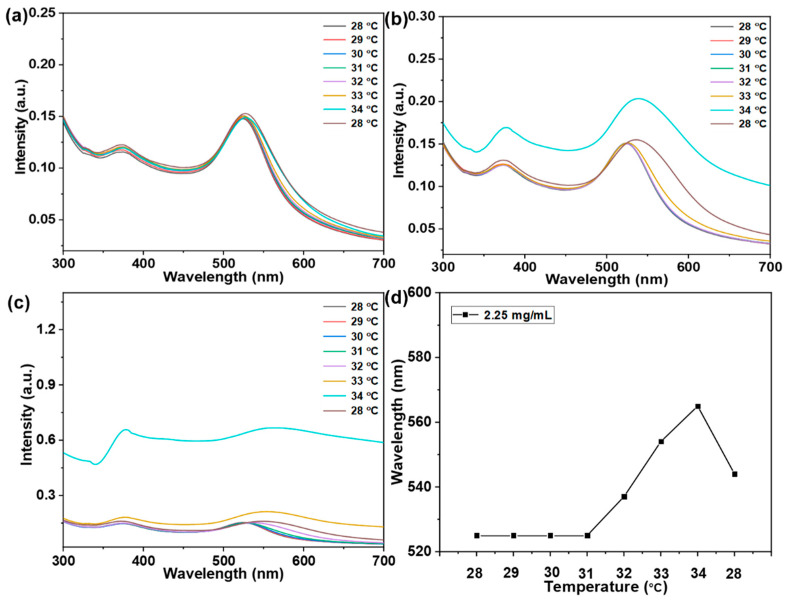
UV–vis absorption spectra of HPEI-IBAm-AuNPs with acylation degree of 50% (**a**) and 65% (**b**) with temperature change; UV–vis absorption spectra (**c**) and maximum absorption wavelengths (**d**) of probes at HPEI-IBAm concentration of 2.25 mg/mL as function of temperature.

Focusing on the narrow range of 32–34 °C for wound temperature changes, we explored the detection conditions of this temperature-responsive probe. First, we compared the effect of HPEI-IBAm at acylation degrees of 50%, 65%, 70%, and 75%, respectively, on temperature detection under the same conditions (Figure 3, Figure S1). The color and turbidity of HPEI-IBAm with a 50% acylation degree did not change significantly during the temperature-increase process. As can be seen from Figure 3a, there was no significant difference in the UV–vis spectrum, indicating that the cloud point of the system at this acylation degree did not match the range of temperature change in the infected wound. In contrast, the HPEI-IBAm systems with 65% (Figure 3b), 70% (Figure S1a,b), and 75% (Figure S1c,d) acylation degrees showed a clear change in the cloud point, which was determined to be 33 °C, 31 °C, and 29 °C, respectively. This clearly reveals a trend of decreasing cloud points of the system with increasing acylation degree. It is particularly noteworthy that the cloud point of the HPEI-IBAm system with a 65% acylation degree falls precisely at 33 °C, which is within the ideal interval for the variation in infected wound temperature. Therefore, based on the above analysis, we decided to select HPEI-IBAm with a 65% acylation degree for subsequent in-depth experimental studies.

**Figure 4:**
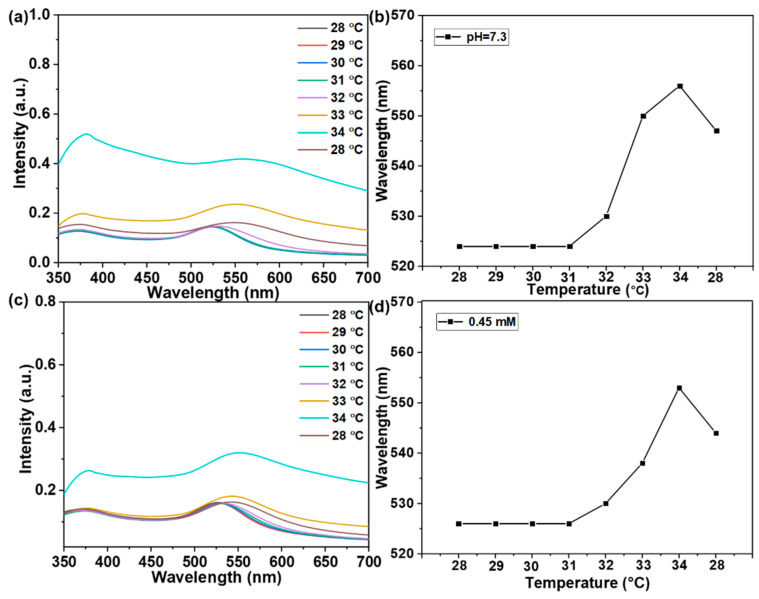
Variation in HPEI-IBAm-AuNP UV–vis absorption spectra (**a**) and maximum absorption wavelengths (**b**) with temperature at pH = 7.3; Variation in probe UV–vis absorption spectra (**c**) and maximum absorption wavelengths (**d**) with temperature at AuNP concentration of 0.45 mM.

In addition, we systematically explored the effects of HPEI-IBAm concentration, AuNP concentration, and pH on the performance of the temperature detection system. Firstly, the HPEI-IBAm concentration was investigated, and the experimental results showed that the cloud point of the system gradually decreased with the increase in HPEI-IBAm concentration. Specifically, when the concentration of HPEI-IBAm was low (Figures S2–S7), the cloud point was high (33, 34 °C), which was beyond the range of temperature variation in infected wounds, and was not unfavorable for accurate detection. Conversely, at excessively high concentrations (Figure S8), the cloud point was too low (30 °C), failing to effectively reflect temperature changes. However, when the concentration of HPEI-IBAm was set at 2.25 mg/mL (Figure 3c), the cloud point was exactly 32 °C, which closely matched to the change in wound temperature and was therefore determined to be the optimal concentration. Secondly, we investigated the effect of AuNP concentration on the detection performance. The data showed that when the concentration of AuNPs was lower than 0.45 mM (Figures S9–S11), the cloud point of the system was high (33 °C), which was unfavorable for detection. However, when the concentration of AuNPs was increased to 0.45 mM, 0.525 mM, and 0.6 mM (Figure 4c, Figures S12 and S13), the cloud point stabilized at 32 °C, which matched the wound temperature. While also being more economical, the concentration of 0.45 mM was selected as the optimal AuNP concentration, as it showed significant changes in the color and UV–vis spectra of the system and was more economical. Finally, we adjusted the pH of the system by changing the NaOH concentration and investigated its effect on the cloud point and detection sensitivity. The experimental results indicated that when the pH value was lower than 7.3 (Figures S14 and S15), the system was insensitive to temperature changes and had a wide detection range. Conversely, when the pH value was higher than 7.3 (Figures S16–S18), the cloud point increased to 33–34 °C, and the change from the clarification to cloud point was rapid, but the changes in the color and UV–vis spectrum were not obvious, which compromised the accuracy of detection. Only at a pH of 7.3 (as shown in Figure 4a,b) was the cloud point of the system 32 °C, accompanied by significant color and UV–vis spectral changes, enabling effective temperature detection. Therefore, we finally determined the optimal pH value to be 7.3.

### 3.3. The Salt Stability of the Probe

**Figure 5:**
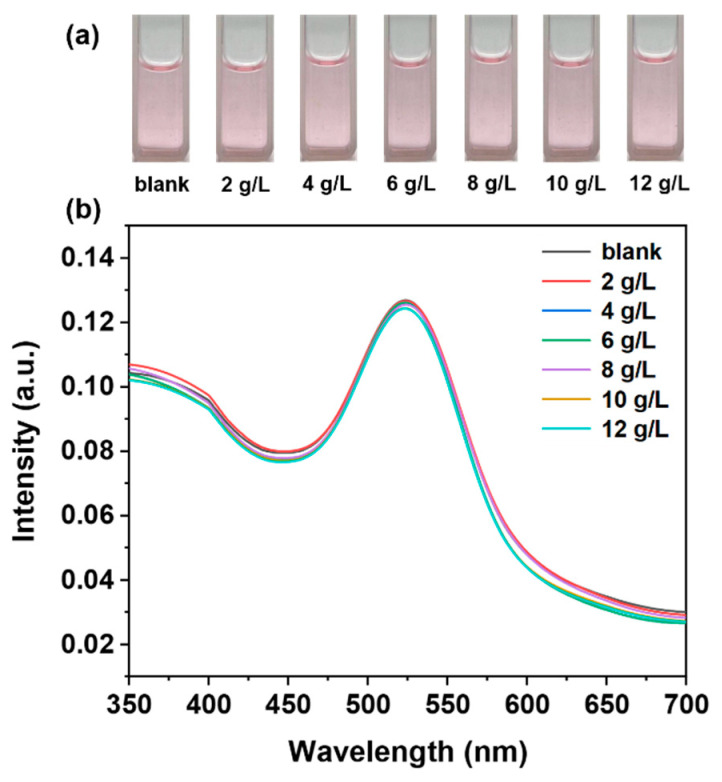
Changes in the photograph of the probe at different salt concentrations (**a**) and changes in its UV–vis absorption spectrum (**b**).

It is well known that AuNP-based probes tend to aggregate under a high ionic strength, resulting in a decrease in the stability of NPs [30], which subsequently impacts their practical applications. During the process of sweating, the salt content in sweat increases significantly. This salty fluid may infiltrate into the system where the probe is located (for instance, when the probe is loaded onto a hydrogel dressing) due to exercise, physical activity, or other physiological factors. Therefore, ensuring the salt resistance stability of the probe is a crucial aspect. The salt resistance stability test results of HPEI-IBAm-AuNPs, presented in Figure 5, showed that the probe was able to remain dispersed without aggregation or precipitation even in a high-salt-concentration environment. This contrasts with the aggregation and color change that traditional AuNPs undergo under high salt concentrations, limiting their applications due to poor salt stability [31]. Furthermore, the probes maintained good stability after three months of storage at room temperature. In summary, AuNPs complexed using HPEI-IBAm exhibit superior stability, attributed to the abundant amino groups in the HPEI-IBAm molecule and its high spatial site resistance effect [32]. The excellent stability of the probe expands its practical application range under various harsh conditions and lays a foundation for its widespread application in the field of temperature detection.

### 3.4. Sensitivity and Reversibility of Temperature Detection by Probes

**Figure 6:**
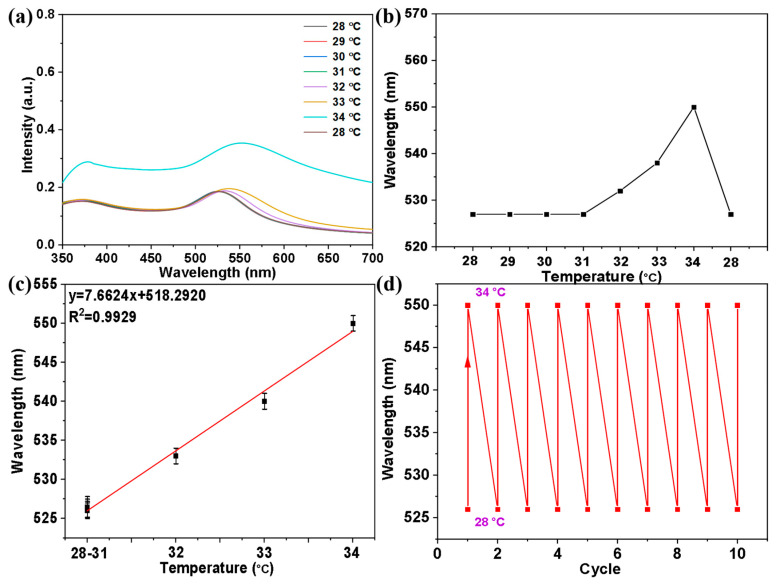
UV–vis absorption spectra (**a**) and maximum absorption wavelength (**b**) versus temperature under optimum conditions for probe; linear relationship between maximum absorption wavelength and temperature under optimum conditions for probe (**c**); and iterative change in maximum absorption wavelength with temperature (**d**).

Under the optimal experimental conditions, the temperature detection behavior of the probe was investigated. Macroscopic observations revealed that as the temperature was increased close to the cloud point of the solution, the color of the solution changed from its original clear pink to a turbid purple. When the temperature was lowered back to the initial level and after a period of waiting, the solution reverted from the turbid purple color to a clear and transparent pink (Figure S19). This phenomenon demonstrates that the polymer has successfully interacted with the AuNPs, enabling reversible aggregation and de-aggregation processes with temperature changes, accompanied by the expression of different color changes. To record this color change process more accurately, we scanned the absorption spectra of the HPEI-IBAm-AuNP composite system at varying temperatures using a UV–vis spectrophotometer (Figure 6a) and analyzed the change in absorption peaks with temperature (Figure 6b). As shown in Figure 6b, as the temperature increases, the absorption spectrum of the solution undergoes a significant red shift, with the maximum absorption wavelength gradually increasing and shifting towards the long-wave direction, which is consistent with the color change shown in Figure S19. When the absorption wavelength is around 526 nm, the light that is complementary to the red light in the incident light is absorbed by the solution, resulting in a pink coloration. However, when the temperature increases beyond the cloud point, the temperature-sensitive polymers shrink and aggregate, causing the AuNPs to aggregate and increase in size. This results in a red shift in the maximum absorption wavelength of the solution, causing the light complementary to violet light in the incident light to be absorbed by the solution, thereby turning it violet. Through the analysis of the above macroscopic and microscopic phenomena, we can confirm that HPEI-IBAm and AuNPs have successfully formed a complex, and this complex exhibit significant color changes with temperature variations. Notably, the probe displayed a good linear relationship within the temperature range of 32–34 °C, with a correlation fitting coefficient as high as 0.9929 (Figure 6c). As shown in Table S1, comparing the detection range of this probe with other reported temperature-sensitive probes, it is clear that this probe has a significant advantage in terms of detection range [23,25,33,34,35]. Furthermore, we have also investigated the recyclability of the probe. As shown in Figure 6d, after undergoing 10 cycles of temperature fluctuations within the range of 28–34 °C, the probe was able to maintain an accurate temperature detection capability with minimal signal deviation, demonstrating its excellent recyclability. In summary, the probe provides a solid theoretical foundation for applications in visual temperature sensing.

### 3.5. Probe Temperature Detection for Real-World Applications

**Figure 7:**
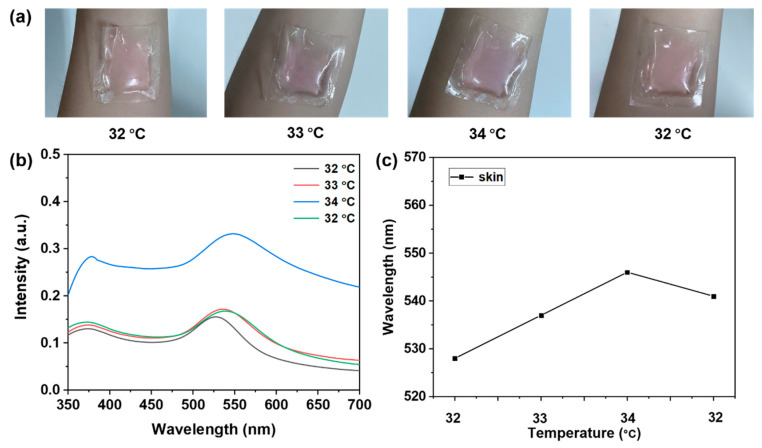
Photographs of probe composite polymer films on skin as a function of temperature (**a**), the UV–vis absorption spectrum (**b**), and the variation in maximum absorption wavelength (**c**) with temperature on skin.

**Figure 8:**
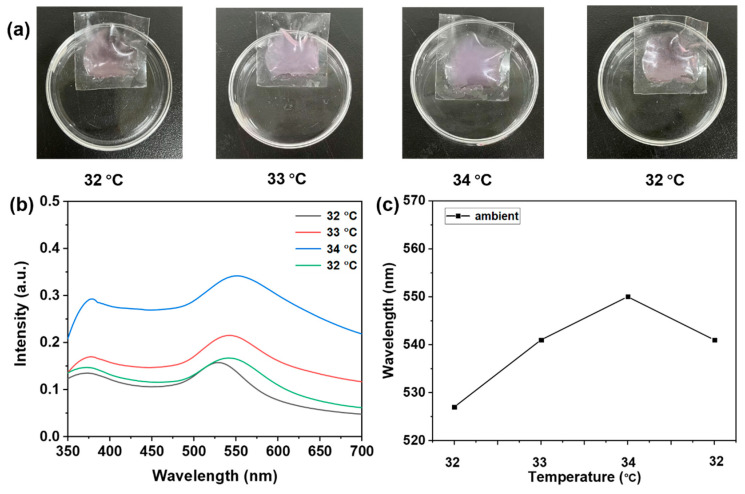
A photograph of the probe composite polymer film in the ambient environment as a function of temperature (**a**), UV–vis absorption spectrum (**b**), and the variation in maximum absorption wavelength (**c**) with temperature in the environment.

To validate the utility of the probe for temperature detection and to further evaluate its precision and accuracy, the probe combined with a PDMS polymer membrane was used for temperature detection for the skin and environment. As shown in Figure 7 and Figure 8, during the change in skin temperature from 32 °C to 34 °C, we could clearly observe that the probe changed from its original clear pink color to a turbid purple color. And when the temperature decreased to 32 °C again, the probe returned to its original color, which not only demonstrates the high sensitivity of the probe, but also reflects its excellent reversibility. In addition, we tested the detection accuracy of the probe. As shown in Table 1, the recoveries of the probe were 99.07%~100.61% for skin temperature detection and 99.10%~100.59% for ambient temperature detection, which once again confirms that the probe maintains a high level of accuracy and reliability in different application scenarios. These results fully demonstrate that the developed probe has high accuracy in temperature detection, laying the foundation for its application in fields such as the temperature detection of infected wounds.

## 4. Conclusions

In this study, highly sensitive temperature-responsive visual probes were designed and prepared based on hyperbranched temperature-sensitive polymers and AuNPs. The morphology and composition of the probe were characterized and analyzed using XPS, TEM, DLS, and NMR. The probe accurately captures and responds to subtle temperature changes within the range of 32–34 °C, accompanied by an obvious color change from clear pink to turbid purple, with a linear fitting coefficient of 0.9929. It is particularly noteworthy that the probe remained stable at salt concentrations up to 12 g/L and that the maximum wavelength of the UV–vis absorption peak remained stable and reversible after 10 cycles of testing. More importantly, a flexible colorimetric temperature sensor can be constructed by combining this probe with PDMS film, and the recoveries of the probe for skin temperature and ambient temperature detection were 99.07%~100.61% and 99.10%~100.59%, respectively. These data strongly verify that the probe can maintain high accuracy and reliability in different application scenarios. In addition, due to its excellent flexibility, elasticity, and ductility, it can be expected to be used for rapid visual detection of temperature in various parts of the human body, presenting favorable application prospects. In summary, the developed HPEI-IBAm-AuNP visual probe can serve as a novel colorimetric method for detecting temperature and is expected to be further applied to the monitoring of wound infections.

## Figures and Tables

**Table 1:** Recovery rate of human skin temperature (1–4) and environment temperature (5–8) detection measured by probe.

Sample	Entry	Probe Detection Temperature (UV–Vis) (°C)	Thermometer Detects Temperature (°C)	Recovery (%)	RSD% (n = 3)
skin	1	32	32.3	99.07	0.8105
	2	33	32.8	100.61	0.7389
	3	34	34.2	99.41	0.5114
	4	32	31.9	100.31	1.0337
environment	5	32	32.2	99.37	1.3351
	6	33	33.3	99.10	0.5881
	7	34	33.8	100.59	0.6632
	8	32	32.1	99.69	0.9483

## Data Availability

Data are contained within the article.

## Supplementary Materials

The following supporting information can be downloaded at https://www.mdpi.com/article/10.3390/s24227124/s1, Figure S1: Variation of probe photographs (a) and UV-vis absorption spectrum (b) with temperature at 70% acylation of HPEI-IBAm-AuNPs; Variation of probe photo (c) and UV-vis absorption spectrum (d) with temperature at 75% acylation of HPEI-IBAm-AuNPs; Figure S2: Variation of probe photographs (a), UV-vis absorption spectra (c) and maximum absorption wavelength (d) with temperature at HPEI-IBAm concentration of 0.75 mg/mL; Figure S3: Variation of probe photographs (a), UV-vis absorption spectra (c) and maximum absorption wavelength (d) with temperature at HPEI-IBAm concentration of 0.75 mg/mL; Figure S4: Variation of probe photographs (a), UV-vis absorption spectra (c) and maximum absorption wavelength (d) with temperature at HPEI-IBAm concentration of 1.25 mg/mL; Figure S5: Variation of probe photographs (a), UV-vis absorption spectra (c) and maximum absorption wavelength (d) with temperature at HPEI-IBAm concentration of 1.5 mg/mL; Figure S6: Variation of probe photographs (a), UV-vis absorption spectra (c) and maximum absorption wavelength (d) with temperature at HPEI-IBAm concentration of 1.75 mg/mL; Figure S7: Variation of probe photographs (a), UV-vis absorption spectra (c) and maximum absorption wavelength (d) with temperature at HPEI-IBAm concentration of 2 mg/mL; Figure S8: Variation of probe photographs (a), UV-vis absorption spectra (c) and maximum absorption wavelength (d) with temperature at HPEI-IBAm concentration of 2.5 mg/mL; Figure S9: Variation of probe photographs (a), UV-vis absorption spectra (b) and maximum absorption wavelength (c) with temperature for AuNPs concentration of 0.225 mM; Figure S10: Variation of probe photographs (a), UV-vis absorption spectra (b) and maximum absorption wavelength (c) with temperature for AuNPs concentration of 0.3 mM; Figure S11: Variation of probe photographs (a), UV-vis absorption spectra (b) and maximum absorption wavelength (c) with temperature for AuNPs concentration of 0.375 mM; Figure S12: Variation of probe photographs (a), UV-vis absorption spectra (b) and maximum absorption wavelength (c) with temperature for AuNPs concentration of 0.525 mM; Figure S13: Variation of probe photographs (a), UV-vis absorption spectra (b) and maximum absorption wavelength (c) with temperature for AuNPs concentration of 0.6 mM; Figure S14: Variation of HPEI-IBAm-AuNPs photographs (a), UV-Vis absorption spectra (b) and maximum absorption wavelength (c) with temperature at pH = 6.9; Figure S15: Variation of HPEI-IBAm-AuNPs photographs (a), UV-Vis absorption spectra (b) and maximum absorption wavelength (c) with temperature at pH = 7.1; Figure S16: Variation of HPEI-IBAm-AuNPs photographs (a), UV-Vis absorption spectra (b) and maximum absorption wavelength (c) with temperature at pH = 7.5; Figure S17: Variation of HPEI-IBAm-AuNPs photographs (a), UV-Vis absorption spectra (b) and maximum absorption wavelength (c) with temperature at pH = 7.7; Figure S18: Variation of HPEI-IBAm-AuNPs photographs (a), UV-Vis absorption spectra (b) and maximum absorption wavelength (c) with temperature at pH = 7.9; Figure S19: Variation of probe photographs with temperature under optimal conditions; Figure S20: Infrared characterisation of HPEI, HPEI-IBAm and HPEI-IBAm-AuNPs; Table S1: Comparison of the temperature detection ranges of several reported visual temperature detection methods. In addition, Table 1 demonstrates the significant advantages of the present probe in terms of performance by comparing the temperature-sensitive probes prepared by previous authors [23,25,33,34,35].
