# Supplementary material for: Hyperbranched Thermosensitive Polymer-AuNP Composite Probe for Temperature Colorimetric Detection
Source: Sensors (Basel). 2024 Nov 6;24(22):7124. doi: 10.3390/s24227124 (PMC11598191; doi:10.3390/s24227124)
Supplement: Supplementary file 1 [file sensors-24-07124-s001.zip › sensors-3287972-supplementary.pdf]

Supporting Information

Huidong Li[1, 2], Yao Zhou[2], Junqi Gu[2], Wenjie Zhong[1, 2], Xinlong Li[1, 2], Xunyong Liu[1, 2, *], Zhuhui Qiao[1, *], and Yi Liu[1, 2, *]

[1] Shandong Laboratory of Yantai Advanced Materials and Green Manufacturing, Yantai 264006, China

[2] School of Chemistry and Materials Science, Ludong University, Yantai 264025, China

*Email: xunyongliu@126.com; Phone /Fax: +86 535 6672176

*Email: zhqiao@licp.cas.cn

*Email: liuyi200541@126.com

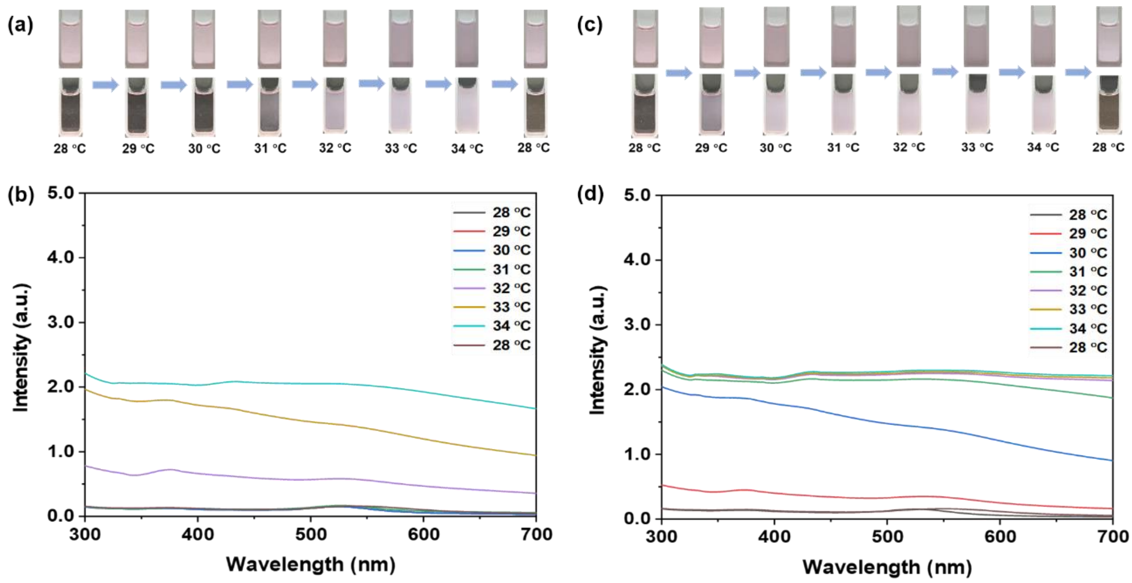

Fig. S1. Variation of probe photographs (a) and UV-vis absorption spectrum (b) with temperature at 70% acylation of HPEI-IBAm-AuNPs; Variation of probe photo (c) and UV-vis absorption spectrum (d) with temperature at 75% acylation of HPEI-IBAm-AuNPs.

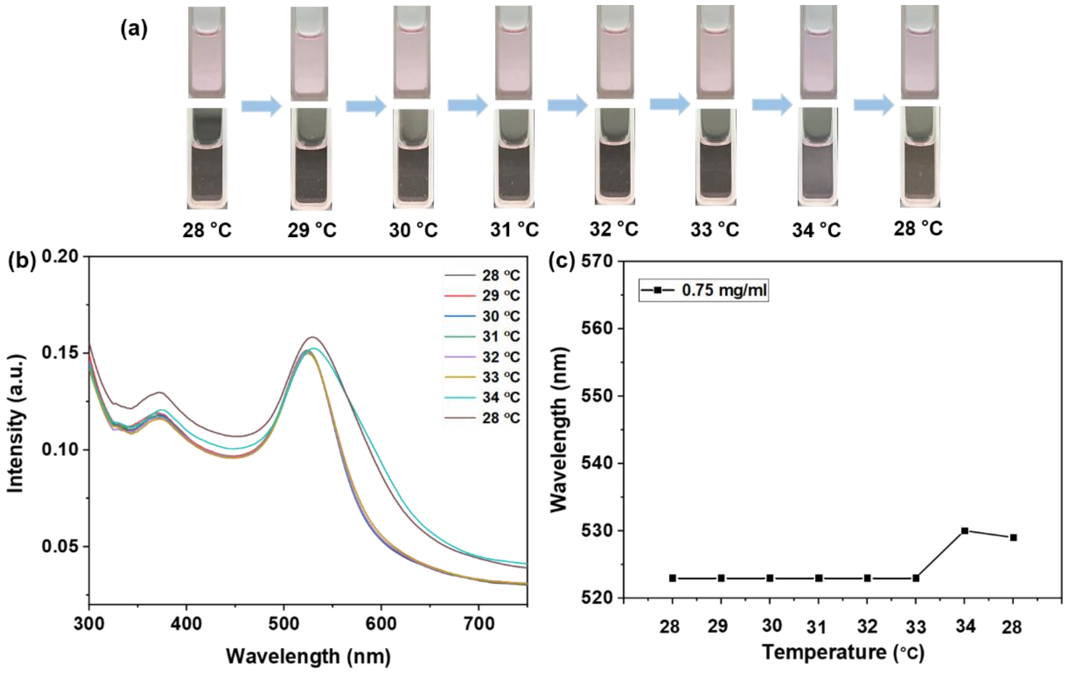

Fig. S2. Variation of probe photographs (a), UV-vis absorption spectra (c) and maximum absorption wavelength (d) with temperature at HPEI-IBAm concentration of 0.75 mg/mL.

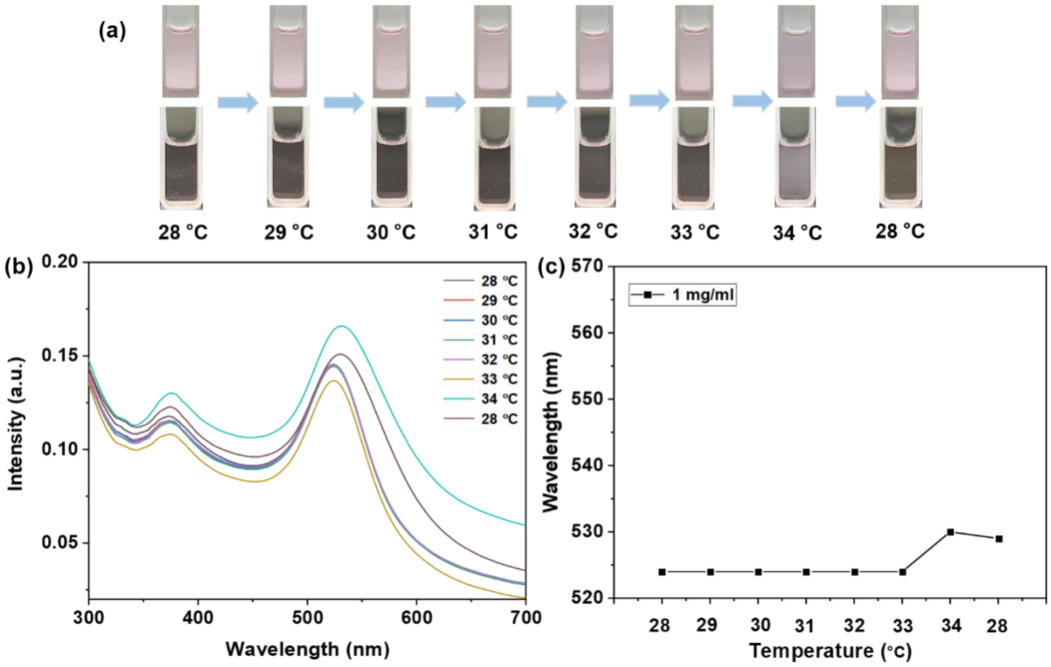

Fig. S3. Variation of probe photographs (a), UV-vis absorption spectra (c) and maximum absorption wavelength (d) with temperature at HPEI-IBAm concentration of 1 mg/mL.

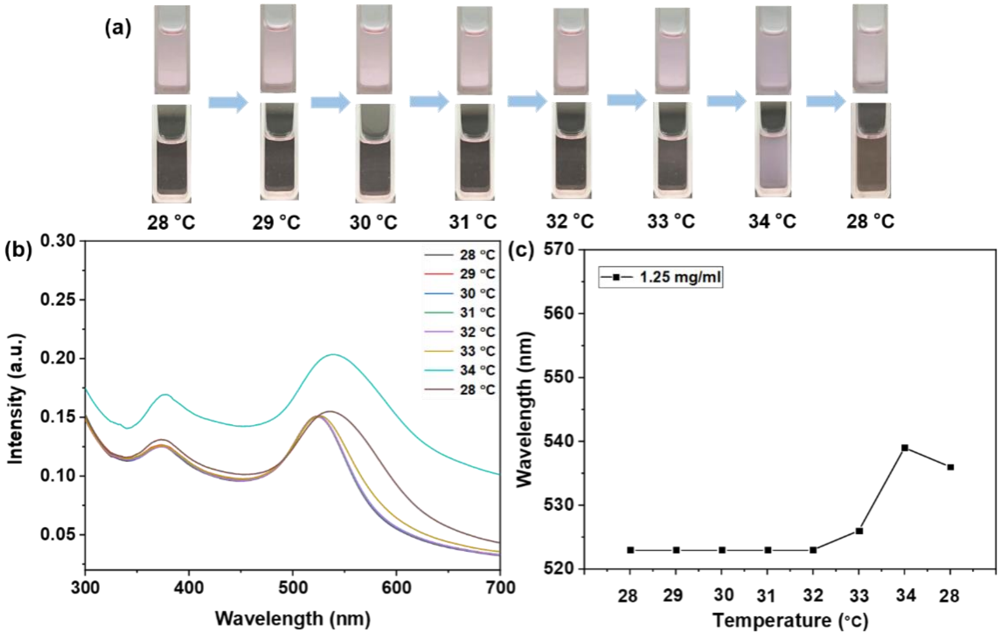

Fig. S4. Variation of probe photographs (a), UV-vis absorption spectra (c) and maximum absorption wavelength (d) with temperature at HPEI-IBAm concentration of 1.25 mg/mL.

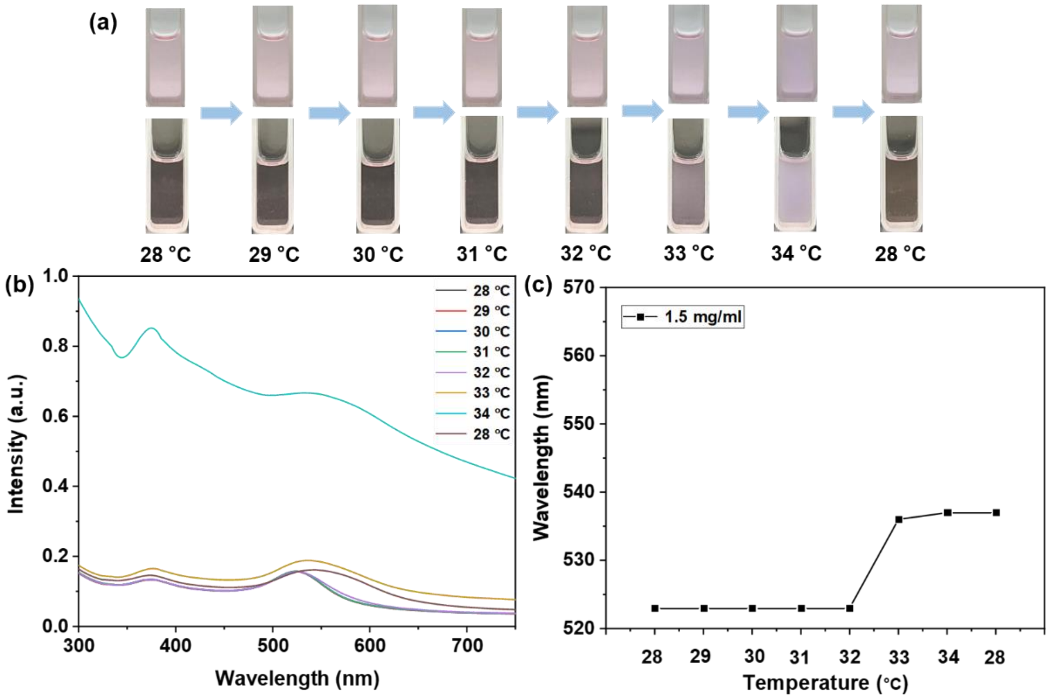

Fig. S5. Variation of probe photographs (a), UV-vis absorption spectra (c) and maximum absorption wavelength (d) with temperature at HPEI-IBAm concentration of 1.5 mg/mL.

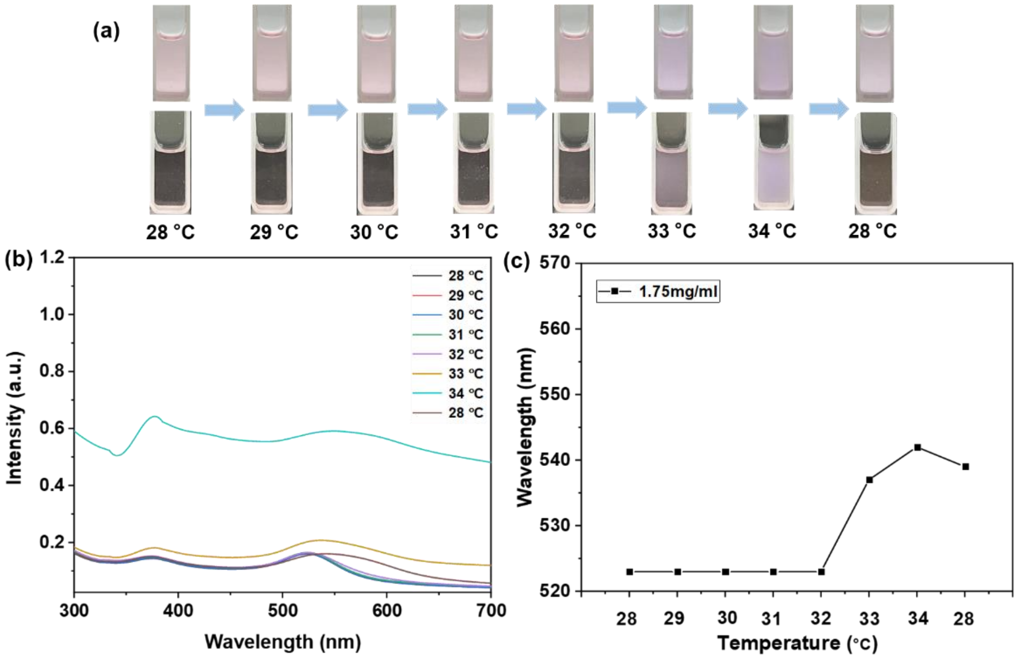

Fig. S6. Variation of probe photographs (a), UV-vis absorption spectra (c) and maximum absorption wavelength (d) with temperature at HPEI-IBAm concentration of 1.75 mg/mL.

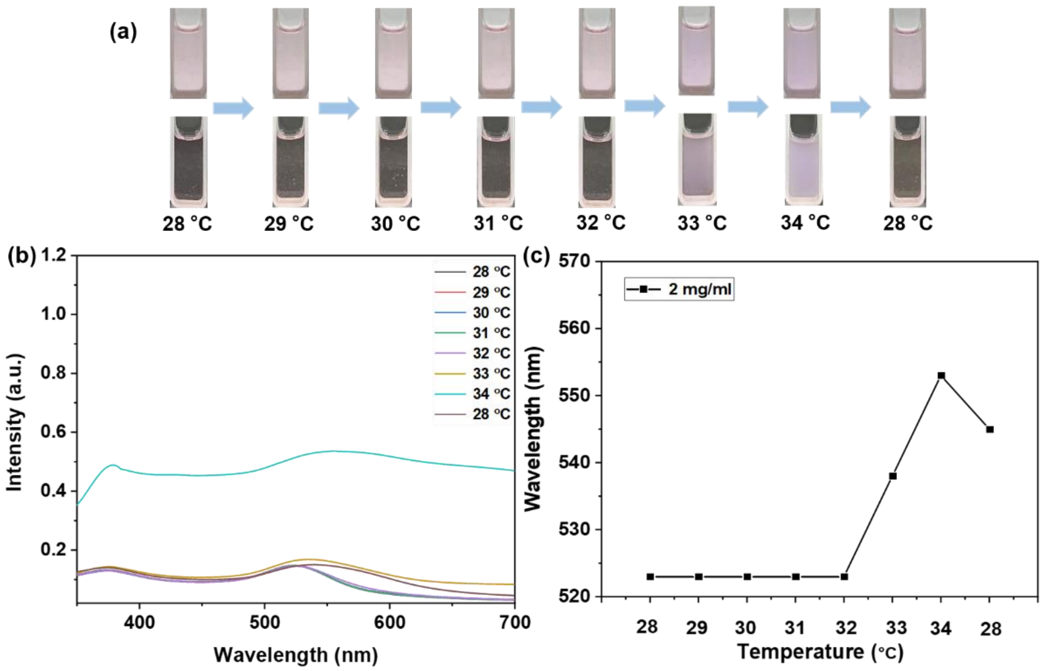

Fig. S7. Variation of probe photographs (a), UV-vis absorption spectra (c) and maximum absorption wavelength (d) with temperature at HPEI-IBAm concentration of 2 mg/mL.

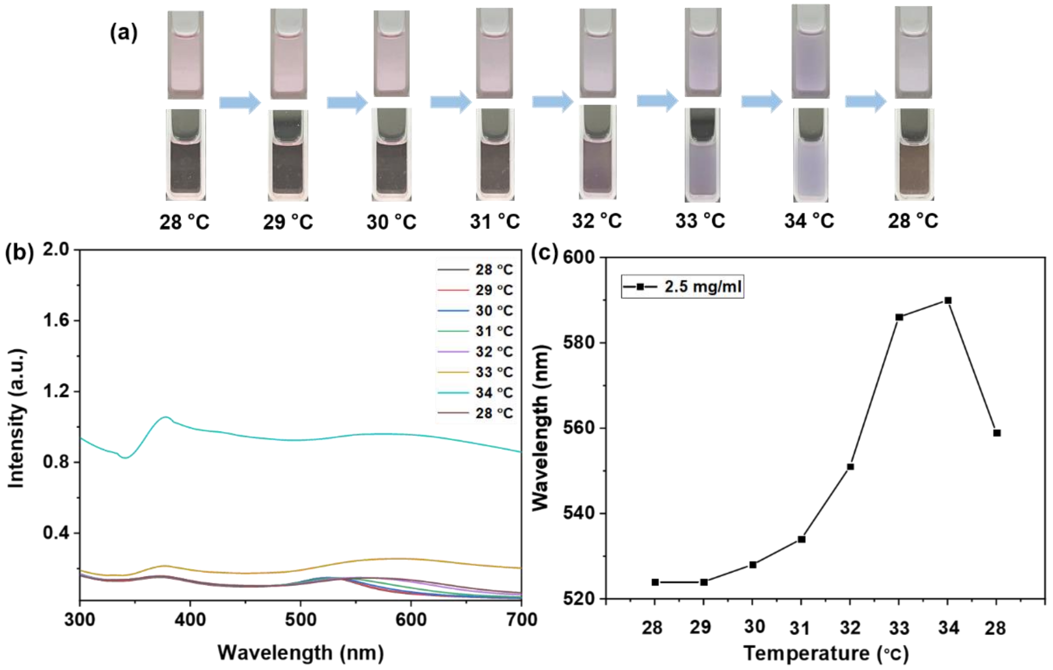

Fig. S8. Variation of probe photographs (a), UV-vis absorption spectra (c) and maximum absorption wavelength (d) with temperature at HPEI-IBAm concentration of 2.5 mg/mL.

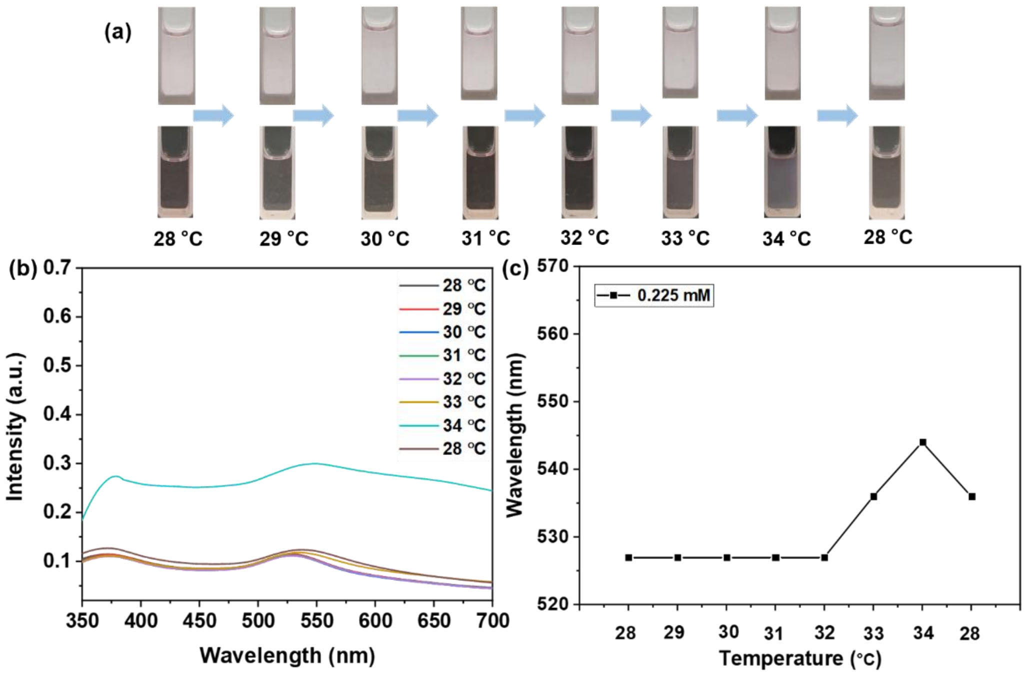

Fig. S9. Variation of probe photographs (a), UV-vis absorption spectra (b) and maximum absorption wavelength (c) with temperature for AuNPs concentration of 0.225 mM.

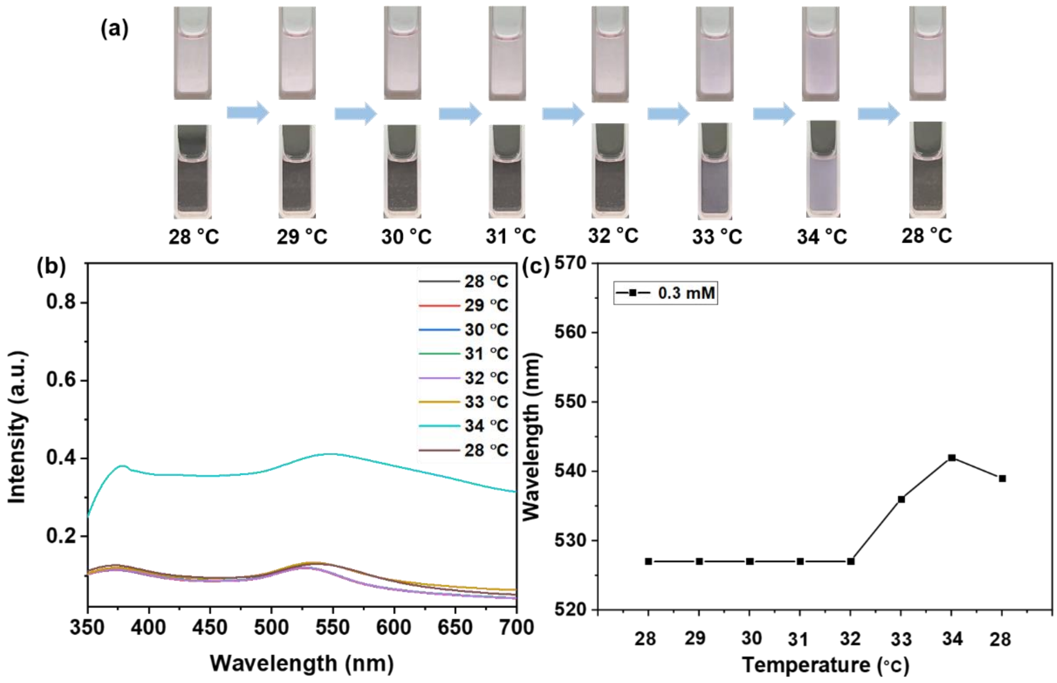

Fig. S10. Variation of probe photographs (a), UV-vis absorption spectra (b) and maximum absorption wavelength (c) with temperature for AuNPs concentration of 0.3 mM.

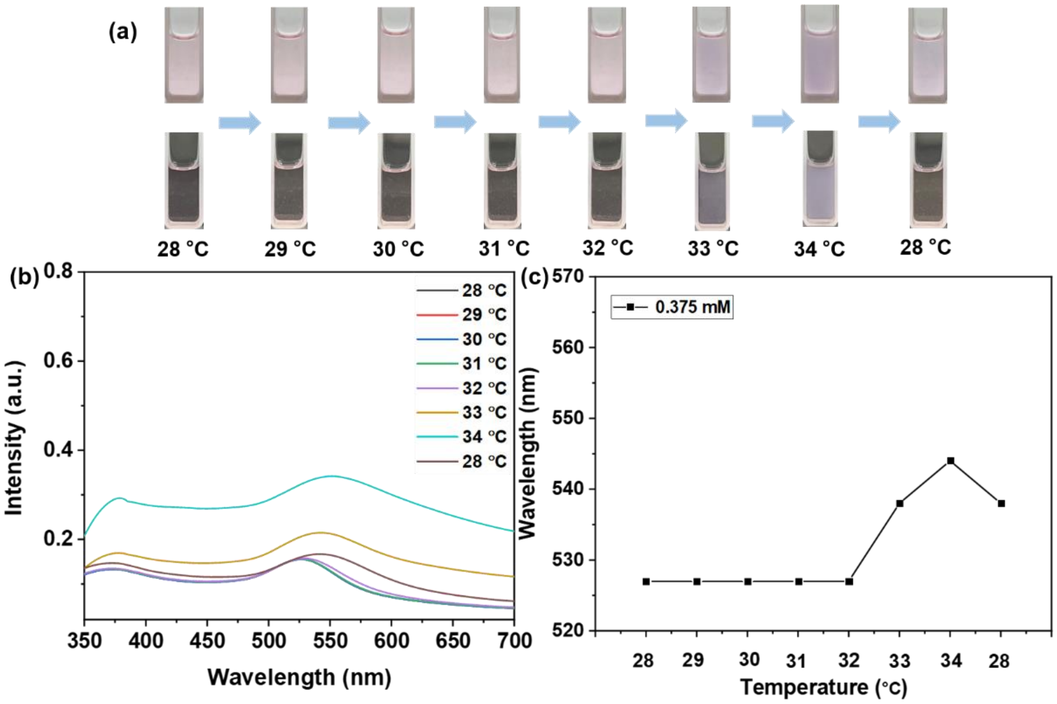

Fig. S11. Variation of probe photographs (a), UV-vis absorption spectra (b) and maximum absorption wavelength (c) with temperature for AuNPs concentration of 0.375 mM.

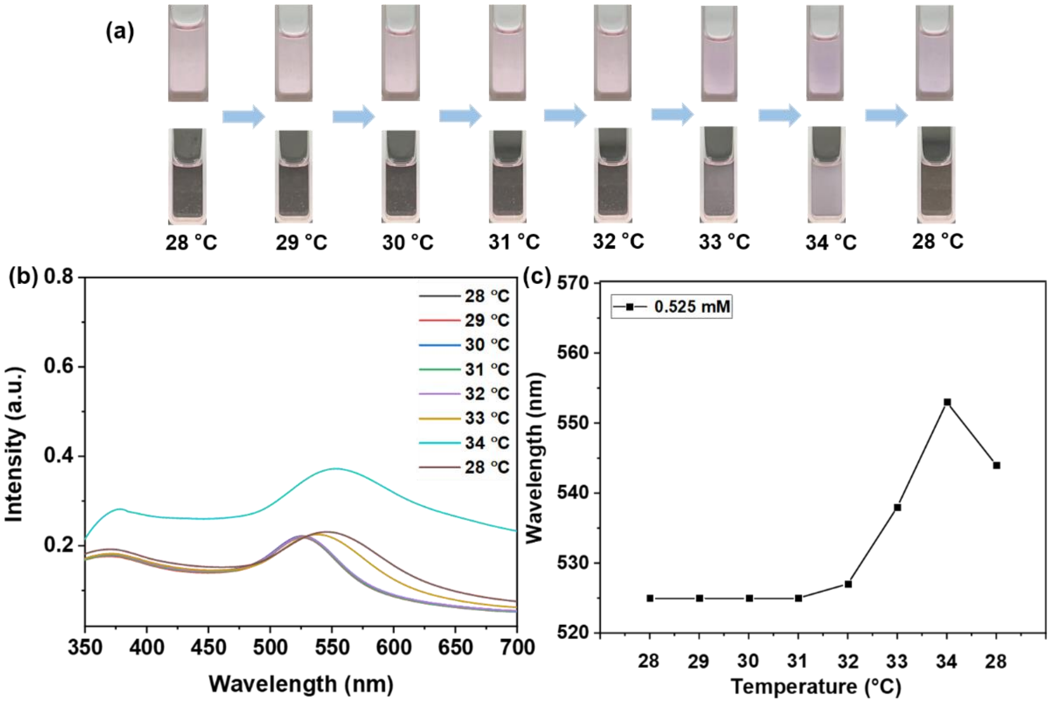

Fig. S12. Variation of probe photographs (a), UV-vis absorption spectra (b) and maximum absorption wavelength (c) with temperature for AuNPs concentration of 0.525 mM.

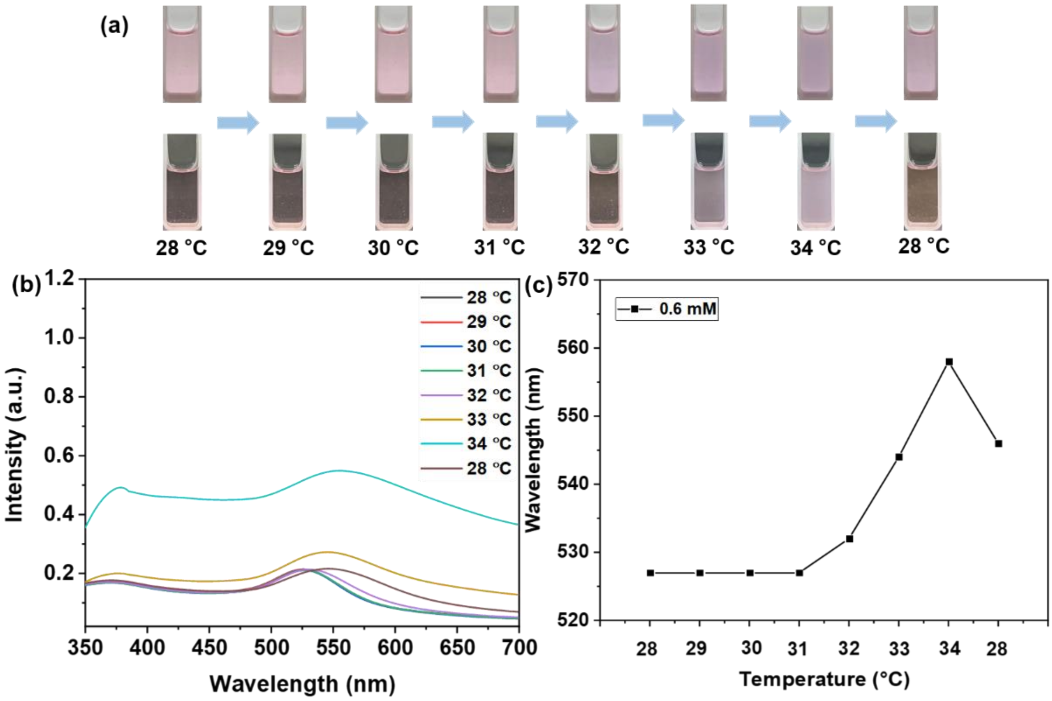

Fig. S13. Variation of probe photographs (a), UV-vis absorption spectra (b) and maximum absorption wavelength (c) with temperature for AuNPs concentration of 0.6 mM.

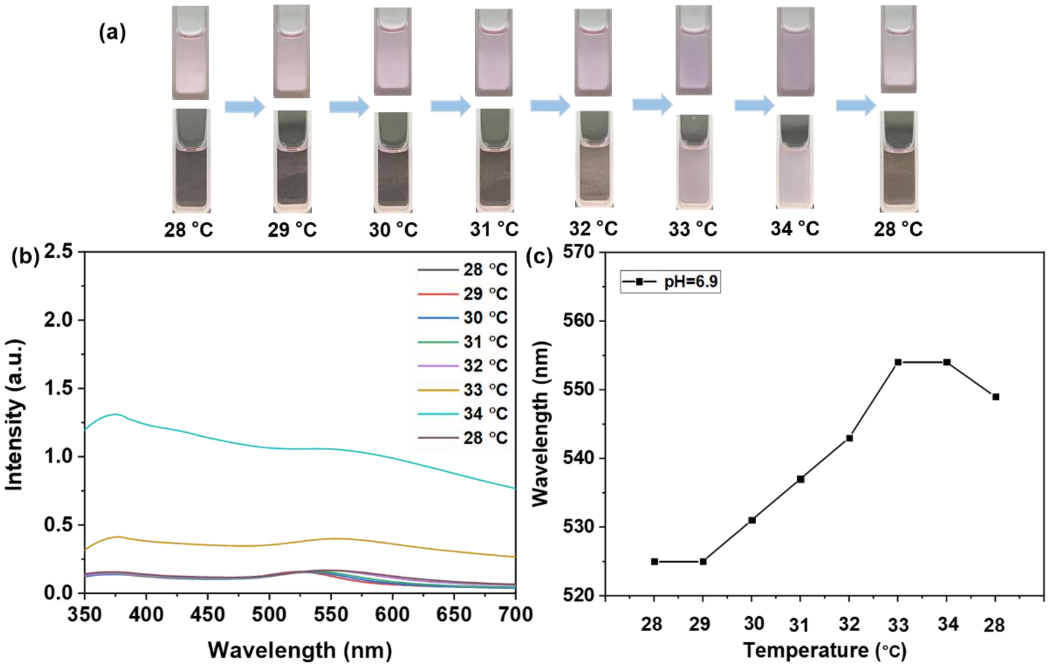

Fig. S14. Variation of HPEI-IBAm-AuNPs photographs (a), UV-Vis absorption spectra (b) and maximum absorption wavelength (c) with temperature at pH=6.9.

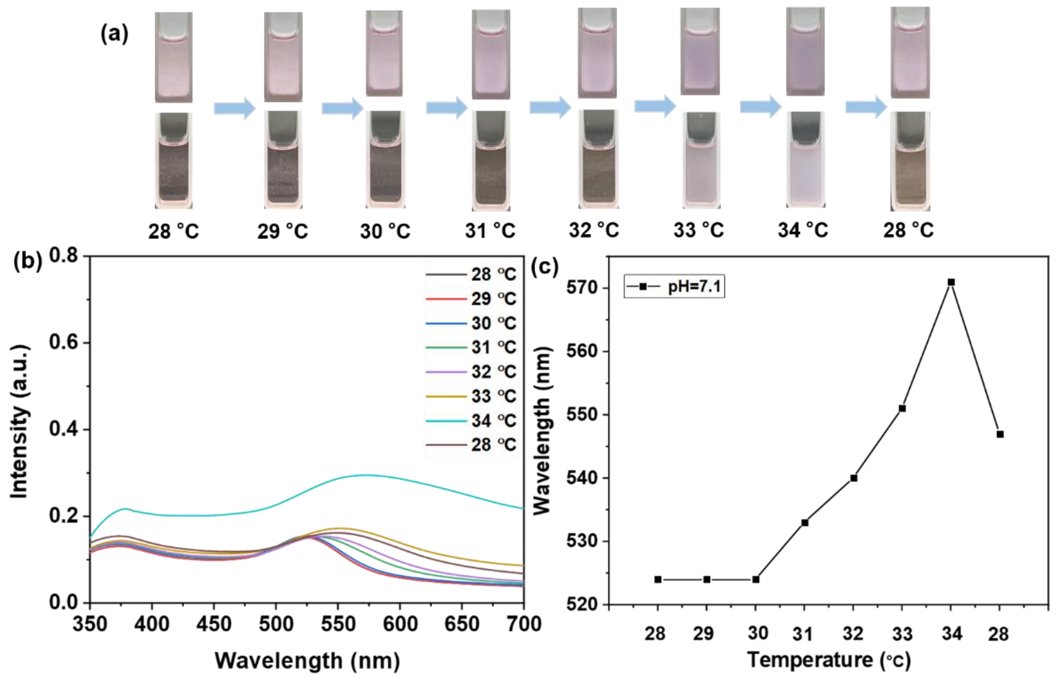

Fig. S15. Variation of HPEI-IBAm-AuNPs photographs (a), UV-Vis absorption spectra (b) and maximum absorption wavelength (c) with temperature at pH=7.1.

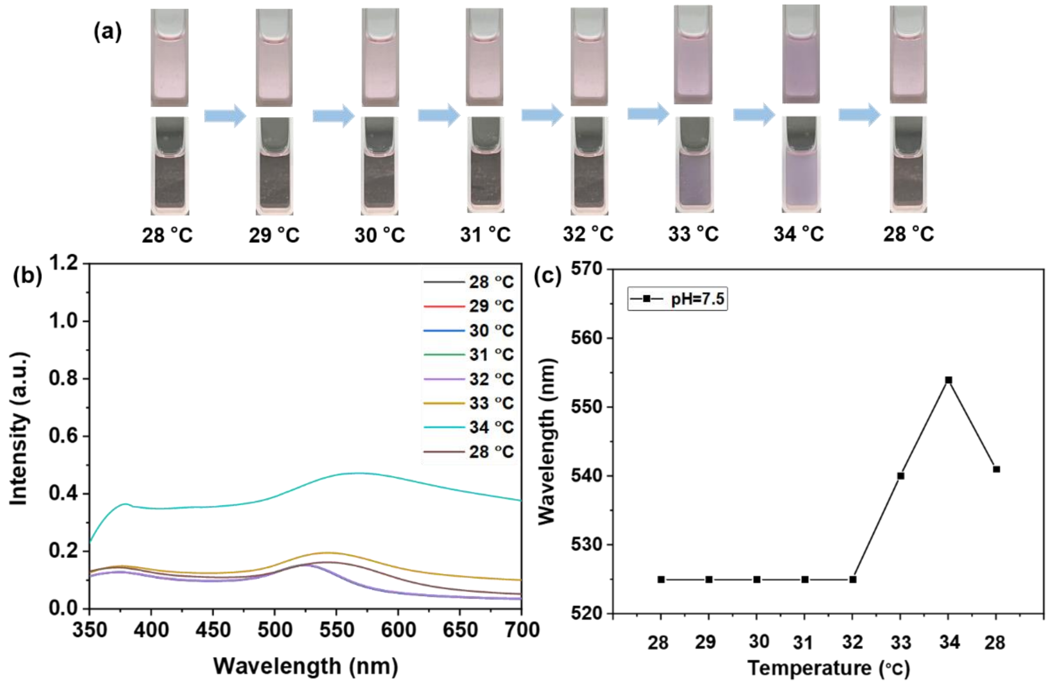

Fig. S16. Variation of HPEI-IBAm-AuNPs photographs (a), UV-Vis absorption spectra (b) and maximum absorption wavelength (c) with temperature at pH=7.5.

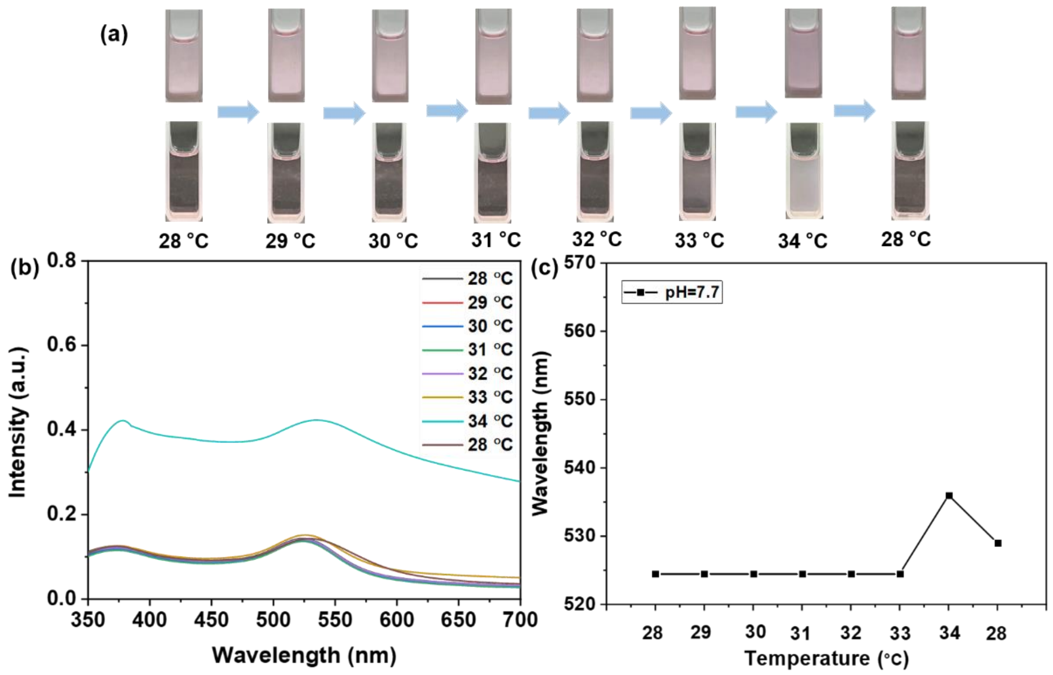

Fig. S17. Variation of HPEI-IBAm-AuNPs photographs (a), UV-Vis absorption spectra (b) and maximum absorption wavelength (c) with temperature at pH=7.7.

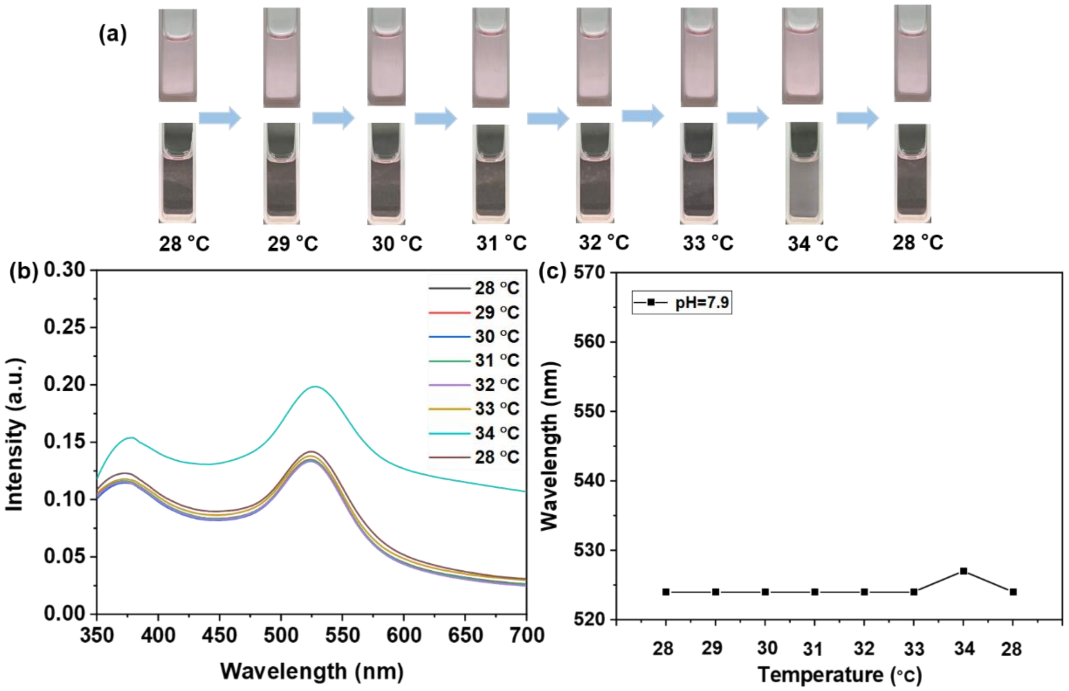

Fig. S18. Variation of HPEI-IBAm-AuNPs photographs (a), UV-Vis absorption spectra (b) and maximum absorption wavelength (c) with temperature at pH=7.9.

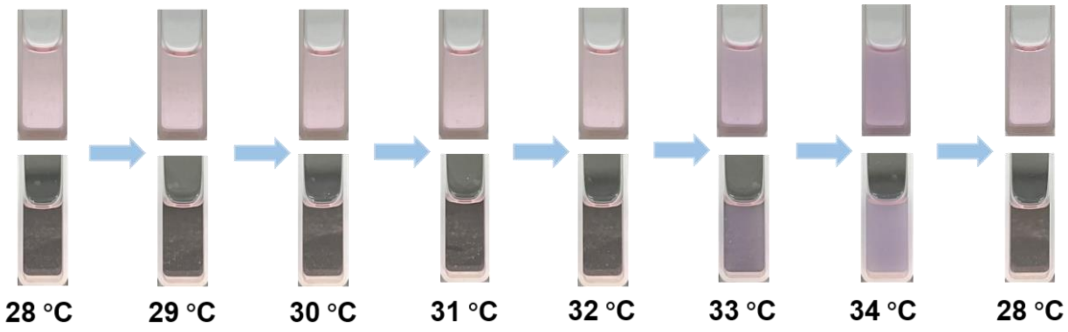

Fig. S19. Variation of probe photographs with temperature under optimal conditions.

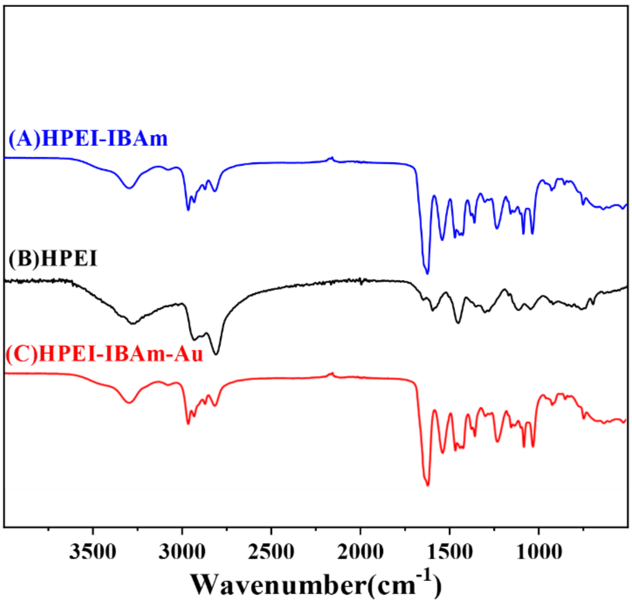

Fig. S20. Infrared characterisation of HPEI, HPEI-IBAm and HPEI-IBAm-AuNPs.

Table S1. Comparison of the temperature detection ranges of several reported visual temperature detection methods.

Probe	Temperature detection interval	References
thermosensitive-AuNPs	5 ℃	[23]
PNIPAM -AuNPs	30 ℃	[25]
CPDs-AgNCs	60 ℃	[33]
silver nanoclusters	40 ℃	[34]
reversible thermochromic fibers	30 ℃	[35]
HPEI-IBAm-AuNPs	2 ℃	**This study**
